# The Ecology of Wrath

**DOI:** 10.3201/eid1910.AC1910

**Published:** 2013-10

**Authors:** Polyxeni Potter

**Affiliations:** Centers for Disease Control and Prevention, Atlanta, Georgia, USA

**Keywords:** art science connection, emerging infectious diseases, art and medicine, Alexandre Hogue, The Ecology of Wrath, coccidioidomycosis, Valley fever, Dust Bowl, American art, about the cover

**Figure:**
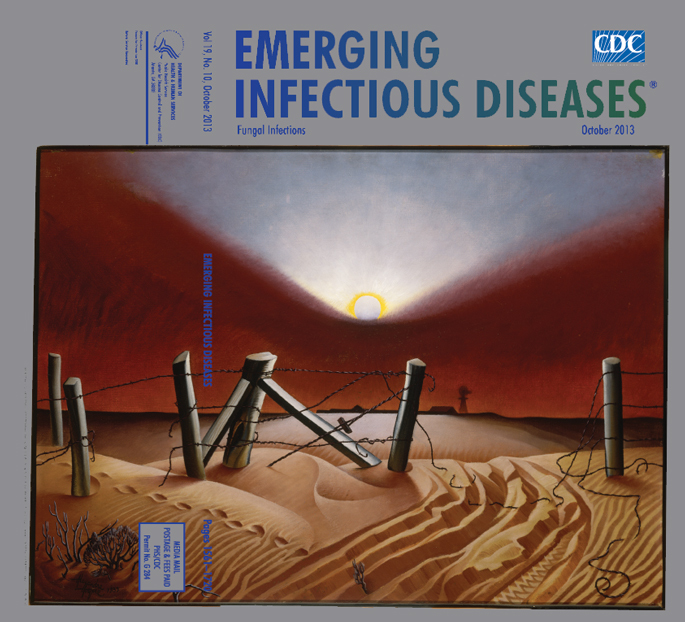
**Alexandre Hogue (1898‒1994) *Dust Bowl* (1933) Oil on canvas (61 cm × 82.8 cm)** Smithsonian American Art Museum, Washington DC, USA, Gift of International Business Machines Corporation, 1969

“Houses were shut tight, and cloth wedged around doors and windows, but the dust came in so thinly that it could not be seen in the air, and it settled like pollen on the chairs and tables, on the dishes,” wrote John Steinbeck in The Grapes of Wrath. The same year, 1939, the author elaborated in a letter that his goal in writing the book was “to rip a reader’s nerves to rags” by laying bare the life of the Dust Bowl migrants with whom he had spent time. Oklahoma Congressman Lyle Boren called the book “an infernal creation of a twisted distorted mind.”

After Congress passed the Homestead Act in 1862, thousands of settlers moved to the semi-arid grasslands of the North American plains to farm and graze cattle. They plowed the fields and planted dryland wheat. High demand generated the promise of economic development and brought in more powerful plows, further expanding arable land. The grasses receded, leaving the ground exposed and vulnerable. When the drought came in 1930, strong winds whipping across the plains created severe dust storms, which continued for nearly a decade, moving millions of tons of topsoil and wiping out farms and ranches across 19 states in the heartland, which became known as the Dust Bowl.

“And then the dispossessed were drawn west―from Kansas, Oklahoma, Texas, New Mexico; from Nevada and Arkansas, families, tribes, dusted out, tractored out. Car-loads, caravans, homeless and hungry; twenty thousand and fifty thousand and a hundred thousand and two hundred thousand,” Steinbeck wrote. Eleanor Roosevelt was less critical of his literary account, “The book is coarse in spots, but life is coarse in spots.”

In 1898, Alexandre Hogue’s family moved from Memphis, Missouri, where he was born, to Denton, Texas, where his life and art would later become inextricably connected with the Dust Bowl. His early copy of Millet’s *The Gleaners*, a painting of peasant women gleaning a field of wheat after the harvest, foreshadowed Hogue’s lifelong interest in rural society and the land. He attended the Minneapolis College of Art and Design and took lessons from Clarence Conaughy and the muralist Lauros Phoenix.

Hogue spent time in New York, absorbing the art scene and forming his own philosophy as painter, lithographer, printmaker, and muralist. “The true artist in painting or any other aesthetic expression sets out to express himself in terms of life he really knows.” He denied lasting influence from any training or external source. “I was considered a dead-on radical when I was young.”

After a 4-year stint in the big city, Hogue became a vocal member of the Dallas Nine and their circle, a group of artists active in the mid-1920s. Other well-known members were Jerry Bywaters, Otis Dozier, Olin Travis, William Lester, Everett Spruce, Thomas Stell, Perry Nichols, Harry Carnohan, and John Douglass. These artists believed in creating their own idiom by portraying the local scene.

Beginning in 1926, Hogue spent time each year in New Mexico, until the beginning of World War II. There he met founding member of the Taos Society of Artists Ernest Blumenschein and American masters Joseph Imhof, Victor Higgins, Emil Bisttram, and Buck Dunton. He made sketching trips into Indian Territory and became acquainted with Indian culture. For creating lasting art, Hogue believed, a feeling for the locality was more important than the natural beauty of the area.

In the 1930s Hogue painted his Erosion series, depicting the devastation of the Dust Bowl, which he witnessed firsthand, “both before and after the dust menace, working and painting on a Panhandle ranch near Dalhart.” It had been “Plowed in on all sides by the ‘suitcase’ farmers, whose uncontrolled loose dirt, pushed before the wind, has gnawed away every sprig of grass that dares show above ground.” When the images appeared in national magazines, reaction was mixed. “Such misinformation will undoubtedly cause tourists and others to abandon or postpone visits to the many important and interesting points in the State of Texas.”

Hogue described his work as “psycho-reality,” involving “mind reactions to real situations, not dreams or subconscious.” He converted his thoughts into abstract visual terms, which were stronger than nature itself. In his work *Drouth Stricken Area*, “The windmill and the drink tub are taken from life,” he wrote. “I worked on that windmill. In fact I was knocked off it by lightning. It was the windmill that was on my sister and brother-in-law’s place―the Bishop Ranch near Dalhart, Texas. The house was strictly my own. I just depicted it so it would be typical of the time…. The placing of a top of a shed coming in front of the tank is strictly a matter of composition. The whole thing is just visually built.”

“Some may feel that in these paintings… I may have chosen an unpleasant subject, but after all the drouth *is* most unpleasant. To record its beautiful moments without its tragedy would be false indeed. At one and the same time the drouth is beautiful in its effects and terrifying in its results. The former shows peace on the surface but the latter reveals tragedy underneath. Tragedy as I have used it is simply visual psychology, which is beautiful in a terrifying way.”

In the *Dust Bowl*, on this month’s cover, the footprints and tire tracks leading away from the farm denote those who fled, the “exodusters.” The angular shapes of fallen fence posts and tangled barbed wire mimic the blood-red wedge of thick dust against the sky. Along the rippled earth, a diminishing truck tire mark suggests that the family has just moved away, their traces already fading in the dust.

More people actually stayed on their land than left, and many died of malnutrition and dust pneumonia―not the only illness eventually associated with the dust. Many discoveries about coccidioidomycosis arose from careful epidemiologic and clinical investigations in California’s San Joaquin Valley during the 1930s, when people migrated there from the Dust Bowl, and during the 1940s when World War II brought military recruits, prisoners of war, and persons of Japanese descent to camps and other areas where the disease is endemic.

Now reemerging, coccidioidomycosis is sometimes called San Joaquin Valley fever or Valley fever. It is caused by a soil fungus found in the southwestern United States, mostly California and Arizona, and in northern Mexico and parts of Central and South America. Dormant during long dry spells, it develops as a mold when the rains come. Spores are swept into the air when the soil is disrupted during construction, farming, earthquakes, or other dust-cloud generating events and are inhaled into the lungs, where the infection begins.

“I don’t like to be called a ‘regionalist’ or ‘American scene painter,’ or, as *Life* magazine called me, ‘painter of the Dust Bowl,” proclaimed Hogue even as he urged farmers to cooperate with federal soil conservation efforts. “My paintings are as much a statement of what may happen as what has happened―a warning of impending danger in terms of present conditions.”

Continued economic development changes the land and draws new populations into disease-endemic areas. In the past decade, state-specific increases in the number of reported coccidioidomycosis cases have been observed in Arizona and in California, where hospitalization costs are increasing. Hogue’s ecologic concerns still apply because, as much as it is vital for creating lasting art, understanding the locality is indispensable for deciphering disease emergence, which is so much a circumstance of time and place. Steinbeck understood the complexity: “The land is so much more than its analysis.”
